# *Ab Initio* Calculations for the Electronic, Interfacial and Optical Properties of Two-Dimensional AlN/Zr_2_CO_2_ Heterostructure

**DOI:** 10.3389/fchem.2021.796695

**Published:** 2021-11-12

**Authors:** Kai Ren, Ruxin Zheng, Junbin Lou, Jin Yu, Qingyun Sun, Jianping Li

**Affiliations:** ^1^ School of Mechanical and Electronic Engineering, Nanjing Forestry University, Nanjing, China; ^2^ School of Information Science and Engineering, Jiaxing University, Jiaxing, China; ^3^ School of Materials Science and Engineering, Southeast University, Nanjing, China; ^4^ School of Automotive and Transportation Engineering, Shenzhen Polytechnic, Shenzhen, China

**Keywords:** first-principles calculation, AlN/Zr_2_CO_2_, type-I band alignment, applications, heterostructure

## Abstract

Recently, expanding the applications of two-dimensional (2D) materials by constructing van der Waals (vdW) heterostructures has become very popular. In this work, the structural, electronic and optical absorption performances of the heterostructure based on AlN and Zr_2_CO_2_ monolayers are studied by first-principles simulation. It is found that AlN/Zr_2_CO_2_ heterostructure is a semiconductor with a band gap of 1.790 eV. In the meanwhile, a type-I band structure is constructed in AlN/Zr_2_CO_2_ heterostructure, which can provide a potential application of light emitting devices. The electron transfer between AlN and Zr_2_CO_2_ monolayer is calculated as 0.1603 |*e*| in the heterostructure, and the potential of AlN/Zr_2_CO_2_ heterostructure decreased by 0.663 eV from AlN layer to Zr_2_CO_2_ layer. Beisdes, the AlN/Zr_2_CO_2_ vdW heterostructure possesses excellent light absorption ability of in visible light region. Our research provides a theoretical guidance for the designing of advanced functional heterostructures.

## Introduction

In 2004, the graphene was prepared and discovered to possess abundant interesting performances (Geim and Novoselov, 2007), which has also encouraged researchers to explore other two-dimensional (2D) materials (Pumera and Sofer, 2017; Sun et al., 2020a; Wang et al., 2020a; Sun and Schwingenschlögl, 2020; Zhang et al., 2021a; Tan et al., 2021) different with bulk materials (Chen et al., 2021). These 2D materials have attracted much attentions because of their unique electronic (Qi et al., 2020), magnetic (Wang et al., 2020b), thermal (Xie et al., 2014; Qin et al., 2019a), mechanical (Qin and Liu, 2017) and optical properties (Wang et al., 2020c). For example, at room temperature, black phosphorus with a thickness of less than 7.5 nm can display transistor performance, and the leakage current modulation order is 10^5^ (Li et al., 2014). Arsenene can adjust its band gap by applying external strain on the surface (Kamal and Ezawa, 2015). Based on transition-metal dichalcogenides (TMDs), PtS_2_, the mobility of field effect transistors (FETs) has been proved to be at least 200 cm^2^/V·s (Pi et al., 2019). All those desirable characteristics promise 2D materials in future advanced applications, such as, photocatalyst (Wang et al., 2019; Zhang et al., 2020a), metal-ion batteries (Sun and Schwingenschlögl, 2021a), and photoelectric devices (Zhang et al., 2020b; Lou et al., 2021).

Recently, in order to further extend the performance and application range of these 2D materials, the prediction of new 2D materials (Sun et al., 2020b; Ding et al., 2020; Sun and Schwingenschlögl, 2021b; Zhang et al., 2021b; Sun et al., 2021) and the modification of known 2D materials have become more and more exciting (Liu et al., 2019; Sun et al., 2019; Zhang et al., 2020c; Wang et al., 2021; Zheng et al., 2021). In many modification methods, two different materials are usually combined as a heterostructure by horizontal (Qin et al., 2019b; Ren et al., 2020a) or vertical direction [(Wang et al., 2020d), (Wang et al., 2020c)]. In particular, the vertical heterostructure is constructed by weak van der Waals (vdW) force at the interface instead of covalent bond, which can result tremendous and novel performances. For example, the type-II heterostructure possesses staggered band alignment, which has ability to separate the photogenerated electrons and holes, revealing a promising application as photocatalyst. It also has been proved by some theoretical and experimental investigations, such as TMDs/BP (Ren et al., 2019a), *h*-BN/C_2_N (Wang et al., 2020e), TMDs/Mg(OH)_2_ (Luo et al., 2019) etc. The type-I band structure in heterostructure can make the charge transfer from wide band gap materials to narrow band gap materials, which can be pretty reflected in light-emitting devices such as LEDs (Bellus et al., 2017; Ren et al., 2021b). Interestingly, the band structure of black/red phosphorus heterostructure can be transformed from type-I to Z-scheme system by quantum confinement effect (Shi et al., 2019). TMDs based heterostructure, such as MoTe_2_/WSe_2_, has excellent photoluminescence (about 1.1 eV from MoTe_2_), which provides promising optoelectronic applications (Yamaoka et al., 2018). Furthermore, type-I heterostructure also can be used as a photocatalyst for water splitting because of remarkable light absorption characteristics (Do et al., 2020; Zhu et al., 2021a). More recently, 2D aluminum nitride (AlN) has attracted significant focus because of novel electronic (Zhang, 2012) and magnetic (Zhang and Zheng, 2011) performances, which also can be tuned by doping (Bai et al., 2015). Besides, some heterostructure constructed by AlN also have been studied, such as BiSb/AlN (Singh and Romero, 2017) and AlN/BP (Yang et al., 2017) etc. Importantly, it has been reported that the AlN films can be prepared on 6H–SiC substrates by various sputtering pressures by RF reactive magnetron sputtering (Kuang et al., 2012) and the AlN nanowires was also has been synthetized (Xu et al., 2003), which demonstrated the preparation method for AlN monolayer. At the same time, the Zr_2_CO_2_ as a MXene materials has been studied extensively to form vdW heterostructure (Zhu et al., 2021b). InSe/Zr_2_CO_2_ heterostructure possesses unique electron mobility (about 10^4^ cm^2^/V·s) as a photocatalyst (He et al., 2019). MoS_2_/Zr_2_CO_2_ heterostructure also has decent band edge positions for the redox reaction of the water splitting (Xu et al., 2020). Interestingly, Zr_2_CO_2_/blue phosphorene heterostructure has a transformable band structure between type-I and type-II under external strain (Guo et al., 2017). Moreover, the MXene also can be prepared by suitable means (Lei et al., 2015). Therefore, both AlN and MXene possess possibility of preparation, which also show the future synthetic work on AlN/MXene heterostructure. And the investigations about the heterostructure based on AlN and Zr_2_CO_2_ monolayer are rare, it is excited to explore the novel properties and the potential application of the AlN/Zr_2_CO_2_ heterostructure.

In this work, the AlN and Zr_2_CO_2_ are selected to build a heterostructure. Using first-principle theoretical calculation methods, the structural and electronic natures of the AlN/Zr_2_CO_2_ heterostructure are addressed, which shows that the type-I band alignment in AlN/Zr_2_CO_2_ heterostructure gives a potential usage of light-emitting devices. Then, the interfacial performances of the heterostructure are calculated by charge density and potential drop. Finally, the light absorption capacity of the AlN/Zr_2_CO_2_ heterostructure is explored.

### Computing Method

In this simulations work, the calculations were performed by first-principles method using density functional theory by the circumstances of Vienna ab initio simulation package (Kresse and Furthmüller, 1996a; Kresse and Furthmüller, 1996b; Capelle, 2006). The generalized gradient approximation and the projector augmented wave potentials were considered to explain the exchange correlation functional (Kresse and Joubert, 1999; Grimme, 2006). Besides, the DFT-D3 method was conducted using Grimme to demonstrate the weak disperson forces (Grimme et al., 2010). Furthermore, the Heyd–Scuseria–Ernzerhof hybrid method was used for decent electronic and optical results of the studied system (Heyd et al., 2005). Moreover, the energy cut-off was 500 eV. The Monkhorst–Pack *k*-point grids was 15 × 15 × 1 and the vacuum space was set as 25 Å, which can efficiently prevent the interaction of nearby layers. The convergence standard for force and energy were limited in 0.01 eV·Å^−1^ and smaller than 0.01 meV, respectively.

## Results and Discussion

**FIGURE 1:**
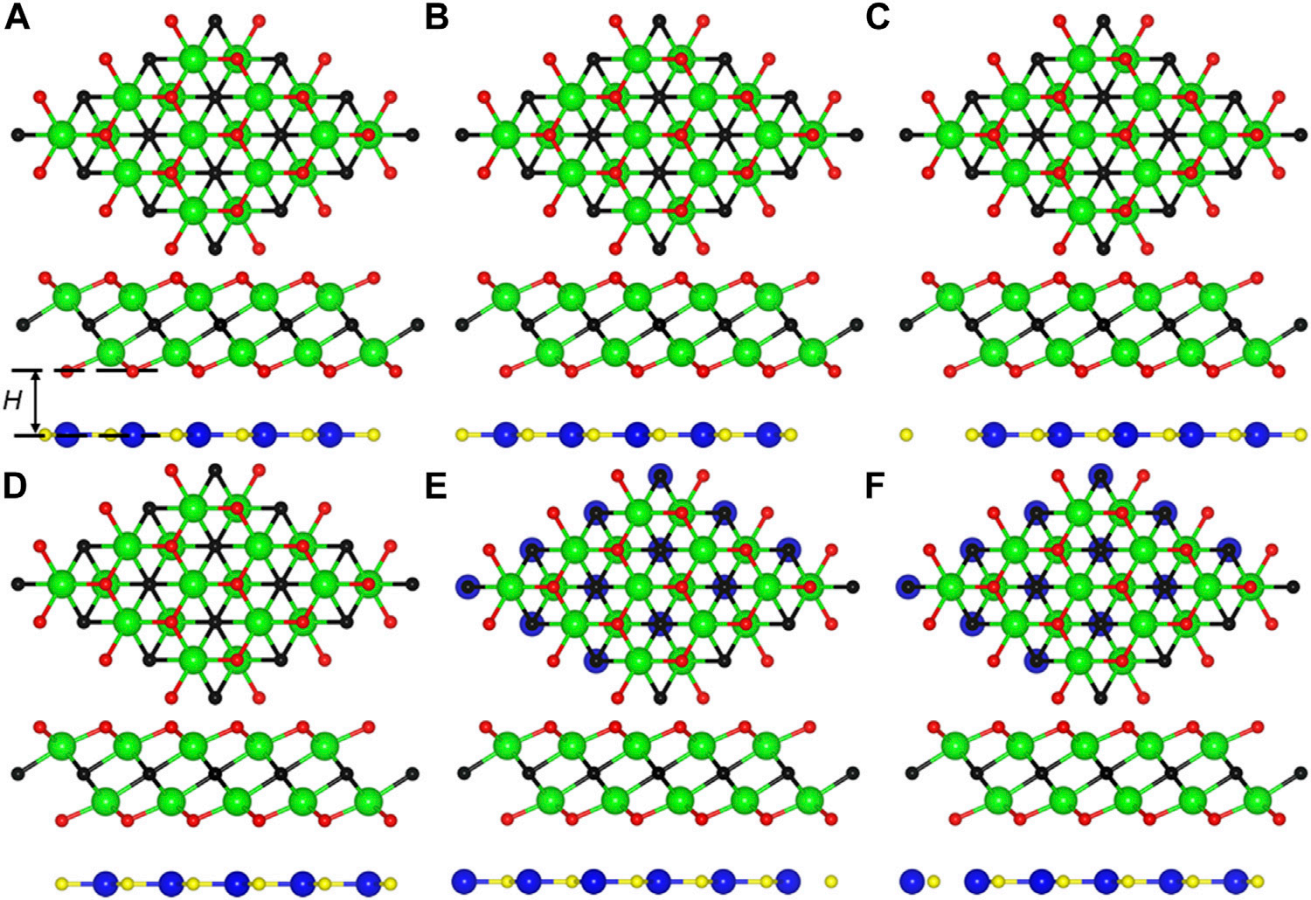
The top and side views of the **(A)** AO-1, **(B)** AO-2, **(C)** AO-3, **(D)** AO-4, **(E)** AO-5, **(F)** AO-6 stacking configurations for the AlN/Zr_2_CO_2_ heterostructure, the blue, yellow, cyan, black and red balls represent the Al, N, Zr, C, and O atoms, respectively.

First, the AlN/Zr_2_CO_2_ is optimized by a decent lattice constant of 3.365 Å, which is comparable with of the AlN (3.127 Å) (Ren et al., 2020b) and Zr_2_CO_2_ (3.294 Å) (Guo et al., 2017) monolayers. When monolayered AlN and Zr_2_CO_2_ are combined to form the heterostructure, considering that there are various combination modes of AlN and Zr_2_CO_2_ monolayers, we only select the most representative highly symmetrical combination configurations among them. These six combination styles of AlN/Zr_2_CO_2_ heterostructure are shown as Figures 1A–F, named AO-1 to AO-6, respectively. For AO-1, the N and Al atoms are located on the upper O and upper Zr atoms, respectively. The AO-2 is obtained by putting the N and Al atoms on the C and lower O atoms, respectively. The AO-3 is built by locating the N and Al atoms on the C and lower Zr atoms, respectively. Then, fixing the N and Al atoms on the lower O and lower Zr atoms, respectively, can construct the AlN/Zr_2_CO_2_ heterostructure by AO-4 configuration. Differently, locating the N and Al atoms on the lower O and C atoms, respectively, can build the AO-5 configuration. Furthermore, AO-6 configuration is constructed by fixing the N and Al atoms on upper O and C atoms, respectively. Besides, the most stable stacking configuration of the AlN/Zr_2_CO_2_ heterostructure is decided by the binding energy, represented by *E*_b_ as follow:

$$E_{b}=E_{AIN/ZrCO2}-E_{AIN}-E_{Zr2CO2}$$ (1)

where *E*_AlN/Zr2CO2_, *E*_AlN_ and *E*_Zr2CO2_ are showing the total energy of the AlN/Zr_2_CO_2_ system, original AlN and Zr_2_CO_2_ monolayers, respectively. Furthermore, the calculation demonstrations that the stacked structure in Figure 1A is the most stable heterostructure with binding energy of –36.05 meV/Å^2^, which also proves that the single-layer AlN and Zr_2_CO_2_ are constructed by vdW force (Chen et al., 2013). In addition, the distance of interface and the bond length of these different stacking configurations of the optimized AlN/Zr_2_CO_2_ heterostructure are calculated in Table 1. Moreover, the following discussion in this work is based on the most stable stacking structure of AO-1.

**TABLE 1:** The optimized distance of interface (*H*, Å) and the bond length (*L*, Å) of the AlN/Zr_2_CO_2_ heterostructure with different stacking styles.

	*H*	*L*_Al–N_	*L*_Zr–C_	*L*_Zr–O_
AO-1	1.909	1.943	2.388	2.110
AO-2	2.231	1.945	2.373	2.139
AO-3	3.235	1.943	2.387	2.137
AO-4	3.626	1.943	2.387	2.136
AO-5	3.475	1.943	2.387	2.137
AO-6	2.710	1.943	2.387	2.131

**FIGURE 2:**
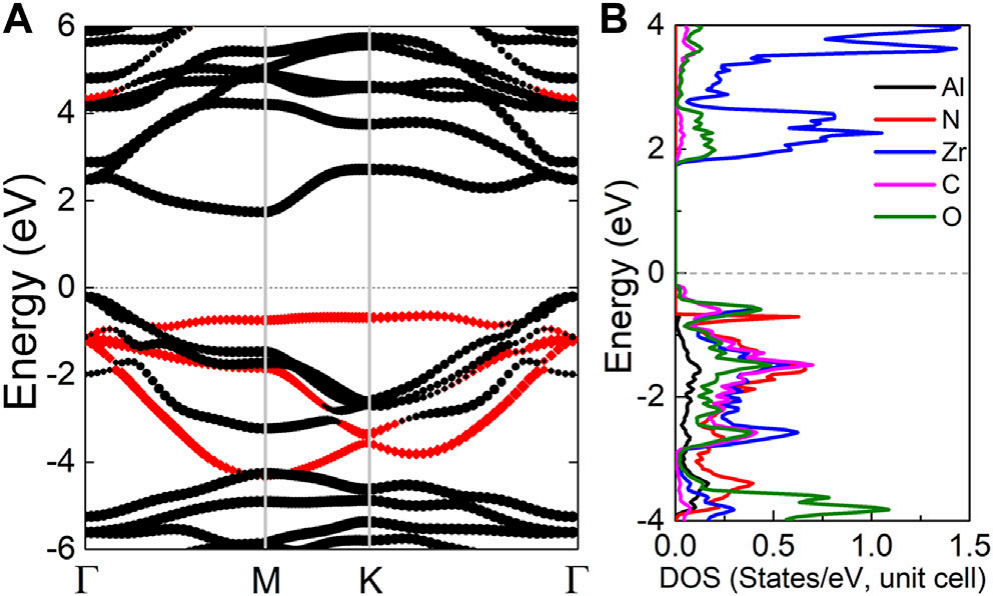
**(A)** The projected band structure and **(B)** the partial density of states of the AlN/Zr_2_CO_2_ vdW heterostructure, the Fermi level is expressed by 0 shown as dash line.

The projected band energy of AlN/Zr_2_CO_2_ vdW heterostructure is obtained by HSE06 calculation, as shown in Figure 2A. One can clearly find that AlN/Zr_2_CO_2_ has a semiconductor nature with indirect band gap of 1.790 eV. In addition, the red and black marks are contributed from AlN and Zr_2_CO_2_ monolayers, respectively, suggesting that the (conduction band minimum) CBM and (the valence band maximum) VBM of AlN/Zr_2_CO_2_ vdW heterostructure are mainly resulted by Zr_2_CO_2_ monolayer. Thus, a type-I band structure is constructed in AlN/Zr_2_CO_2_ vdW heterostructure. Besides, the partial density of AlN/Zr_2_CO_2_ vdW heterostructure, as shown in Figures 2B, 2is also obtained to further prove the characteristics of intrinsic type-I band structure. It is obvious that the CBM and the VBM of the AlN/Zr_2_CO_2_ vdW heterostructure are mainly donated by Zr and O atoms, respectively.

**FIGURE 3:**
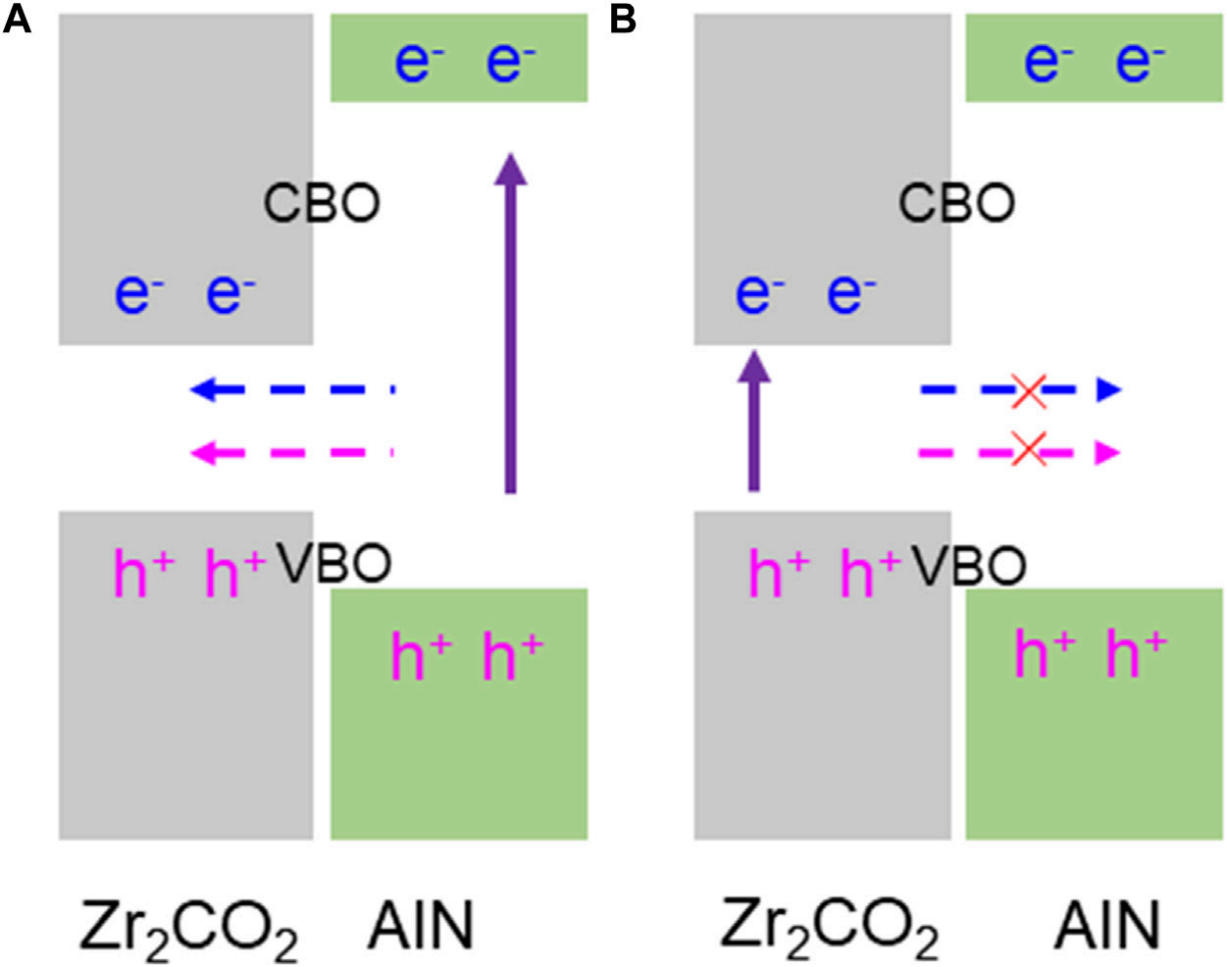
Band alignment schematic of the excited charge transfer mode of the AlN/Zr_2_CO_2_ vdW heterostructure: **(A)** feasible and **(B)** limited charge migration mode.

Such type-I band structure in the AlN/Zr_2_CO_2_ vdW heterostructure provides some important advanced applications in nano-devices. In AlN/Zr_2_CO_2_ vdW heterostructure, as shown in Figure 3A, CBM and VBM of AlN/Zr_2_CO_2_ vdW heterostructure are contributed by single-layer Zr_2_CO_2_, and the band gap of single-layer Zr_2_CO_2_ is less than that of single-layer AlN. When AlN/Zr_2_CO_2_ vdW heterostructure is excited by some external conditions, the electrons in the broad-band gap AlN monolayer are inspired and transferred to the CBM, generating holes at the VBM. It is worth noting that under the action of conduction band offset, CBO (valence band offset, VBO), electrons (holes) at the CBM (VBM) of the AlN layer can be transferred to the CBM (VBM) of the Zr_2_CO_2_ layer. Besides, the obtained CBO and VBO in AlN/Zr_2_CO_2_ vdW heterostructure are 2.432 and 0.471 eV respectively. While the electrons and holes excited in the relatively narrow-band gap of Zr_2_CO_2_ layer cannot be transferred to AlN layer due to low energy, in Figure 3B, which explains the AlN/Zr_2_CO_2_ vdW heterostructure can be considered as a potential light-emitting device material.

**FIGURE 4:**
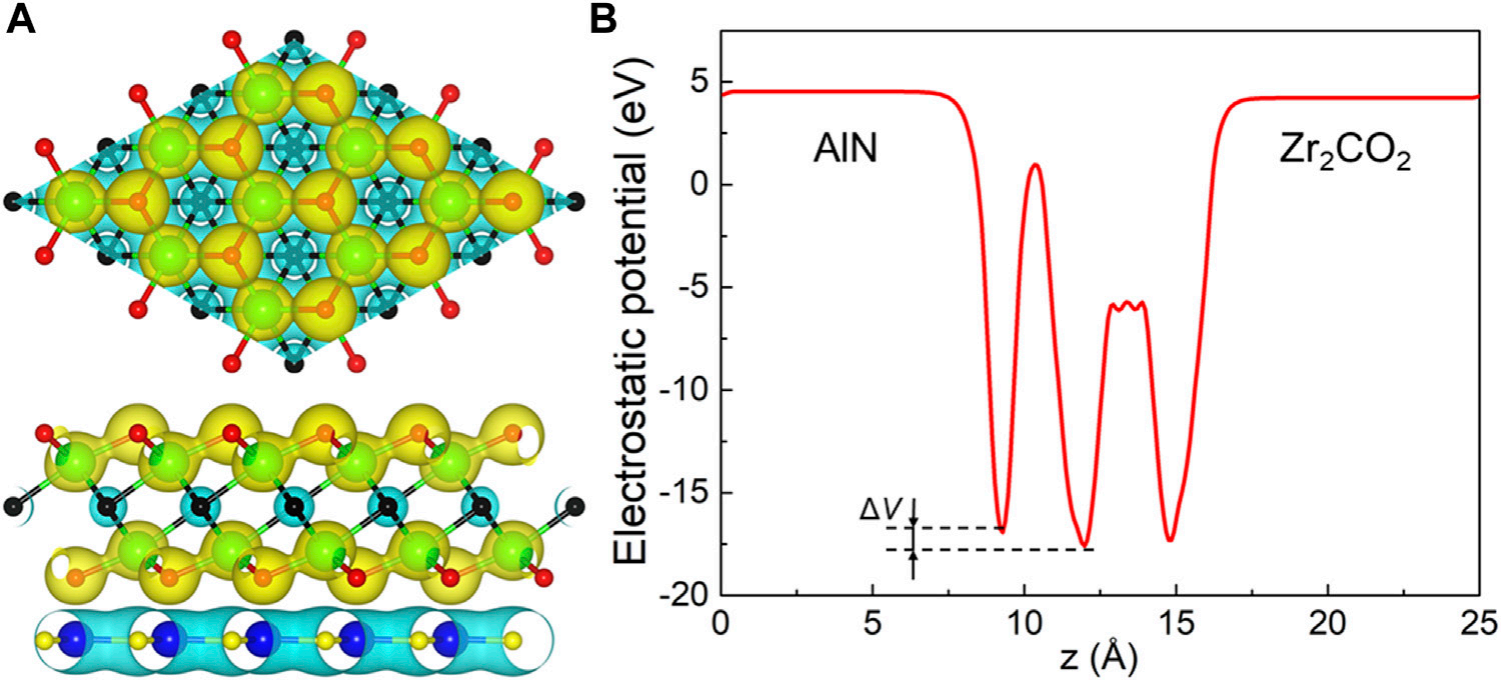
**(A)** The charge density difference and the **(B)** potential drop of the AlN/Zr_2_CO_2_ vdW heterostructure. The yellow and cyan regions contribute the gaining and losing of electrons, respectively.

Then, we discussed the interface properties of AlN/Zr_2_CO_2_ vdW heterostructure by the charge density difference (Δ*ρ*) and the potential drop (Δ*V*) in the interface. The charge density difference across the interface of the AlN/Zr_2_CO_2_ vdW heterostructure is calculated by:

$$\Delta \rho =\rho_{AIN/Zr2CO2}-\rho_{AIN}-\rho_{Zr2CO2},$$ (2)

where *ρ*_AlN/Zr2CO2_, *ρ*_AlN_ and *ρ*_Zr2CO2_ represent the charge density of the AlN/Zr_2_CO_2_ vdW heterostructure, monolayered AlN and Zr_2_CO_2_, respectively. Demonstrated by Figure 4A, the Zr_2_CO_2_ layer acts as an electron acceptor and AlN is an electron donor layer. Through Bader charge analysis (Tang et al., 2009), the obtained charge transfer from AlN layer to Zr_2_CO_2_ layer is 0.1603 |*e*| in AlN/Zr_2_CO_2_ vdW heterostructure. Importantly, there is a certain degree of potential drop across the interface of the AlN/Zr_2_CO_2_ vdW heterostructure, shown as Figure 4B, and the calculated potential drop of 0.663 eV also play a critical role to assist the migration of the excited electrons and holes between the AlN/Zr_2_CO_2_ vdW heterostructure (Wang et al., 2018).

**FIGURE 5:**
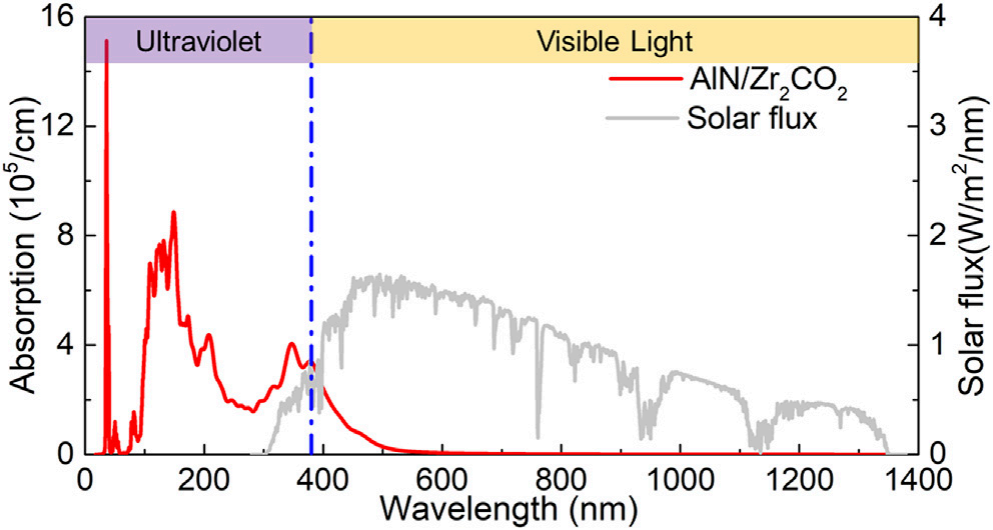
The light absorption spectrum of the AlN/Zr_2_CO_2_ vdW heterostructure.

In order to produce more efficient light-emitting device, active materials should be able to effectively absorb light in the visible and near-infrared regions, especially type-I heterostructure. Therefore, we investigated the light absorption performance of AlN/Zr_2_CO_2_ vdW heterostructure by the calculation:

$$\alpha \left(\omega \right)=\frac{\sqrt{2}\omega }{c}{\left\{{\left[\varepsilon_{1}^{2}\left(\omega \right)+\varepsilon_{2}^{2}\left(\omega \right)\right]}^{1/2}-\varepsilon_{1}\left(\omega \right)\right\}}^{1/2},$$ (3)

where *ω* is the angular frequency; *α* shows absorption coefficient and *c* is the speed of light. Besides, $\varepsilon_{1}\left(\omega \right)$ is used to explain the dielectric constant for real parts, which the imaginary one is demonstrated by $\varepsilon_{2}\left(\omega \right)$ . As shown in Figure 5 (the data source of solar flux is obtained from NREL website), AlN/Zr_2_CO_2_ vdW heterostructure demonstrates capacity to absorb sunlight over a wide range in the visible region the AlN/Zr_2_CO_2_ vdW heterostructure possesses a lot of absorption peaks. In ultraviolet region (left side of blue dotted line), the AlN/Zr_2_CO_2_ vdW heterostructure exhibits an absorption peak of 3.97 × 10^5^ cm^−1^at the wavelength as 344 nm. In the in the visible region (right side of blue dotted line), the obtained absorption peak is 3.14 × 10^5^ cm^−1^ locating at the wavelength of 369 nm, which is higher than other studied 2D heterostructures, such as WS_2_/GeC (2.651 × 10^5^ cm^−1^) (Ren et al., 2021a), Arsenene/GaS (1.403 × 10^5^ cm^−1^) (Li et al., 2021), g-GaN/BSe (1.470 × 10^5^ cm^−1^) (Ren et al., 2019c) etc. The calculated results demonstrate the AlN/Zr_2_CO_2_ vdW heterostructure possesses excellent light absorption capacity.

## Conclusion

In conclusion, the AlN and Zr_2_CO_2_ monolayers are constructed by vdW force to form a heterostructure. And the most stable AlN/Zr_2_CO_2_ is decided by the lowest binding energy of about –36.05 meV/Å^2^. The, the HSE06 obtained projected band structure shows the AlN/Zr_2_CO_2_ vdW heterostructure possesses semiconductor nature with a band gap of 1.790 eV, and presents a type-I energy band alignment, which is a satisfaction candidate for light-emitting devices. Furthermore, the interface characteristics of AlN/Zr_2_CO_2_ vdW heterostructure is investigated by charge density difference (0.1603|*e*| from AlN layer to Zr_2_CO_2_ layer) and potential drop (0.663 eV). Moreover, the AlN/Zr_2_CO_2_ vdW heterostructure explains a remarkable light absorption performance, which can further offer excellent technical guidance for nano light-emitting device materials.

## Data Availability

The raw data supporting the conclusions of this article will be made available by the authors, without undue reservation.
